# The PD-1/PD-L1 inhibitory pathway is altered in pre-eclampsia and regulates T cell responses in pre-eclamptic rats

**DOI:** 10.1038/srep27683

**Published:** 2016-06-09

**Authors:** Mei Tian, Yonghong Zhang, Zhaozhao Liu, Guoqiang Sun, Gil Mor, Aihua Liao

**Affiliations:** 1 Family Planning Research Institute, Center for Reproductive Medicine, Tongji Medical College, Huazhong University of Science and Technology, Wuhan 430030, P.R. China; 2 Department of Obstetrics and Gynecology, Maternal and Child Health Hospital of Hubei province, Wuhan, P.R. China; 3 Department of Obstetrics, Gynecology & Reproductive Sciences, Division of Reproductive Sciences, Yale University School of Medicine, New Haven, CT 06520, USA

## Abstract

The programmed cell death-1(PD-1)/PD-ligand 1 (PD-L1) pathway is critical to immune homeostasis by promoting regulatory T (Treg) development and inhibiting effector T (such as Th17) cell responses. However, the association between the PD-1/PD-L1 pathway and the Treg/Th17 imbalance has not been fully investigated in pre-eclampsia (PE). In this study, we observed an inverse correlation between the percentages of Treg and Th17 cells, and the expression of PD-1 and PD-L1 on the two subsets also changed in PE compared with normal pregnancy. We further explored their relationship *in vivo* using the L-NG-Nitroarginine Methyl Ester (L-NAME) induced PE-like rat models, also characterized by Treg/Th17 imbalance. Administration of PD-L1-Fc protein provides a protective effects on the pre-eclamptic models, both to the mother and the fetuses, by reversing Treg/Th17 imbalance through inhibiting PI3K/AKT/m-TOR signaling and enhancing PTEN expression. In addition, we also observed a protective effect of PD-L1-Fc on the placenta by reversing placental damages. These results suggested that altered PD-1/PD-L1 pathway contributed to Treg/Th17 imbalance in PE. Treatment with PD-L1-Fc posed protective effects on pre-eclamptic models, indicating that the use of PD-L1-Fc might be a potential therapeutic target in PE treatment.

Pre-eclampsia (PE) is a pregnancy-specific, immune-mediated syndrome affecting approximately 2–7% of pregnant women, a primary cause of maternal and perinatal mortality globally^1^,^2^. Although efforts have been made, there is still a void in understanding its clear pathogenesis. Due to the life-threatening risk of PE and the lack of effective treatment, there is a pressing need for us to identify the key pathogenesis of PE and find effective treatment to protect both the mothers and babies.

Balanced immune responses are essential for the maintenance of successful pregnancy^3^. Aberrant responses of the immune system during pregnancy are suggested to play an important role in the pathogenesis of PE^4^. Various immunological factors, such as activated monocytes and neutrophil, dysfunctional cytokines, T helper -1 pre-dominance over Th2 cells and imbalance between regulatory T (Treg) and Th17 cells etc., have been reported in PE^5^,^6^,^7^,^8^. Treg cells are a specialized subset of T cells, with the suppressive capacity and regulatory function, playing an important role in the induction of maternal tolerance to the fetus and the maintenance of normal pregnancy (NP)^9^,^10^,^11^. Their absence impairs mice pregnancy, while the adoptive transfer of Treg cells, not only could rescue pregnancy in abortion-prone mice^12^, but also reduces IL-17 increased abortion rates in the CBA/J× BALB/c mouse model^13^. Therefore, the balance between Treg and Th17 cells plays a critical role in the establishment of maternal-fetal tolerance and maintenance of pregnancy. Numerous studies proved that elevated levels of Treg cells are associated with NP^14^, while deficiencies in quantity and/or function of Treg cells and/or excessive Th17-immunity have been demonstrated in women suffering from PE^15^,^16^,^17^. What contributes to a Treg/Th17 imbalance in PE has not been ascertained.

The interaction between programmed cell death-1 (PD-1 or CD279) and its ligand (PD-L1 or CD274) has emerged as a key player in regulating immune response and peripheral tolerance^18^,^19^,^20^. The PD-1/PD-L1 pathway defends against potentially pathogenic effector T cells by simultaneously harnessing two mechanisms of peripheral tolerance: (I) promoting Treg development and function and (II) directly inhibiting pathogenic effector T cells^20^. PD-1-deficient mice developed spontaneous autoimmunity diseases, such as arthritis, lupus-like glomerulonephritis and cardiomyopathy^21^,^22^,^23^. Engagement of PD-1 with PD-L1 negatively regulates Th17 cells, which play pathogenic roles in the development of autoimmune diseases and graft-versus-host disease (GVHD)^24^,^25^. Apart from autoimmune disorders, this pathway has been proven to be involved in the establishment of maternal-fetal tolerance^26^,^27^, since PD-L1 blockade results in the reduction in litter size, number and increase in embryo resorption of mice, and the failure of fetal–maternal tolerance with Treg deficiency and hyperactivity of Th17 cells^28^. Therefore, the altered PD-1/PD-L1 pathway may be associated with the Treg/Th17 imbalance in human pregnancy^29^,^30^. The PD-1/PD-L1 pathway is considered a particularly attractive therapeutic target in autoimmune diseases, because the development of PD-1 agonists could deliver the necessary ‘one-two punch’ to protect against self-reactivity: (I) augmenting iTreg function and (II) concomitantly suppressing the expansion and functions of activated effector T cells^20^. Administration of soluble PD-L1-Fc protein has been reported to diminish the severity of collagen-induced arthritis and T-cell induced chronic colitis in the mouse model and to inhibit cell proliferation and production of IL-17 and IL-23 by splenocytes^24^,^31^,^32^. Decreased Treg numbers and increased Th17 activation are associated with PE. However, whether the PD-1/PD-L1 pathway is of relevance for Treg/Th17 imbalance in PE has not been explored and is the main aim of our study.

## Results

### The Treg/Th17 imbalance and altered PD-1 and PD-L1 expression on the two subsets were observed in women with PE

**Figure 1:**
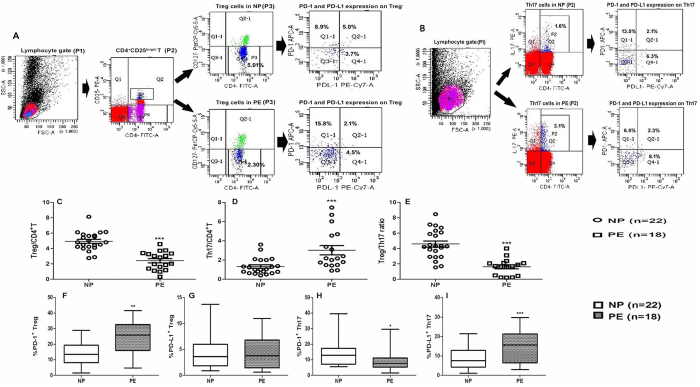
Gating strategies and repertoire of Treg and Th17 cells in peripheral blood of pre-eclamptic women. **(A)** Representative flow cytometry analyses of peripheral blood Treg cells stained with mAbs to CD4, CD25 and CD127. Dot plots for PD-1-APC and PD-L1-PE-Cy7 staining of Treg cells were made. (**B**) Representative flow cytometry analyses of peripheral blood Th17 cells stained with mAbs to CD4 and IL-17A. Dot plots for PD-1-APC and PD-L1-PE-Cy7 staining of Th17 cells were made. The percentages of various subsets are indicated in each quadrant. **(C)** The percentages of Treg cells significantly decreased in PE. **(D)** The percentages of Th17 cells significantly increased in PE. **(E)** The Treg/Th17 cell ratios were significantly lower in PE than in NP. **(F)** PD-1 expression on Treg cells in PE was higher than that in NP. **(G)** No difference was found in the PD-L1 expression on Treg cells between NP and PE. **(H)** PD-1 expression on Th17 cells in PE was lower than that in NP. **(I)** PD-L1 expression on Th17 cells in PE was higher than that in NP. Data are presented as median (range) in (**C–E)**. Data are presented as the Mean ± SEM in (**F–I**). *P < 0.05, **P < 0.01, ***P < 0.001 vs NP. NP, normal pregnancy; PE, pre-eclampsia.

Our first objective was to characterize the percentages of circulating Treg cells and Th17 cells in blood samples from women with NP and women with PE (Table 1). Treg cells and Th17 cells were defined as CD4^+^ CD25^bright^CD127^low/−^ and CD4^+^ IL-17A^+^, respectively. Gating strategies for Treg cells and Th17 cells were shown in Fig. 1A,B. The percentages of each subpopulation were determined by the proportion of either among CD4^+^ T cells. We observed an inverse correlation between the percentages of Treg and Th17 cells in NP versus PE. In PE, the percentage of Treg cells significantly decreased (*P* = 0.000) (Fig. 1C), and the percentage of Th17 cells increased (*P* = 0.000) (Fig. 1D) compared with NP. Moreover, the Treg/Th17 cell ratios were lower in PE than those in NP (*P* = 0.000) (Fig. 1E).

The PD-1/PD-L1 pathway is critical to the development and function of immune cells. Therefore, we hypothesized that the decrease of the number of Treg cells found in PE patients might be associated with changes in the expression and/or function of the PD-1/PD-L1 axis. To test this hypothesis, we first evaluated the expression levels of PD-1 and PD-L1 in circulating Treg and Th17 cells in women with NP and PE. Gating strategies for PD-1 and PD-L1 expression on Treg and Th17 cells were shown in Supplementary Figs S1 and S2.

Our data showed differential expression of PD-1 between the two groups. Thus, the percentage of PD-1^+^ Treg cells was higher in PE than in NP (*P* = 0.001) (Fig. 1F). However, no difference was found in the percentage of PD-L1^+^ Treg cells (*P* = 0.877) (Fig. 1G) between NP and PE. That was not the case when we evaluated the expression of PD-1 and PD-L1 in Th17 cells. The percentage of PD-1^+^ Th17 cells was lower (*P* = 0.022) (Fig. 1H), while the percentage of PD-L1^+^ Th17 cells increased in PE, compared with NP (*P* = 0.020) (Fig. 1I). This data suggests a potential correlation between the expression of PD-1/PD-L1 and the ratios of Treg/Th17 cells in NP and PE.

### Treatment with PD-L1-Fc ameliorates L-NAME-induced PE-like symptoms in rat models

**Figure 2:**
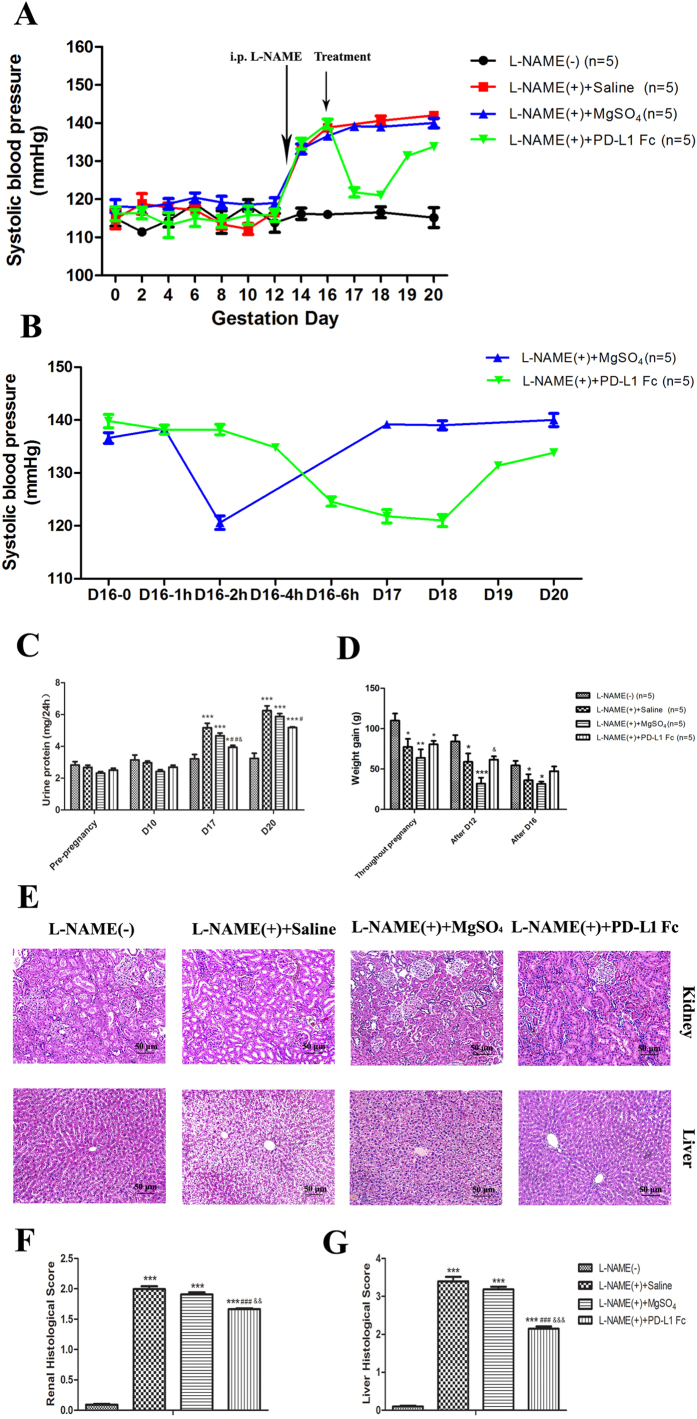
Effects of PD-L1-Fc on PE-like symptoms in pre-eclamptic rats. **(A)** SBP of the four groups was evaluated pre- and post-pregnancy. **(B**) The fluctuations of SBP in the groups of L-NAME (+) +MgSO_4_ and L-NAME (+) +PD-L1-Fc from GD16 to delivery were more closely observed. **(C)** The 24-h urinary protein levels of the four groups before pregnancy, on GD 10, GD17 and 20 were analyzed. **(D)** The net weight gain of each pregnant rat was observed throughout pregnancy (the difference between GD21 body weight and GD2 body weight) and after GD12 (the difference between GD21 body weight and GD12 body weight) and GD16 (the difference between GD21 body weight and GD16 body weight). **(E)** The structural changes of kidneys and livers of four groups were observed by HE staining (×200). **(F)** Renal tissues were scored for the histological damages. **(G)** Histological scores of liver tissues of each group. Data are presented as Mean ± SEM. GD 0 = the day before pregnancy; *P < 0.05, **P < 0.01, ***P < 0.001 vs. L-NAME (−) group; ^#^*P* < 0.05, ^###^*P* < 0.001 vs. L-NAME (+) group; ^&^*P* < 0.05, ^&&^*P* < 0.01, ^&&&^*P* < 0.001 vs. L-NAME (+) +MgSO_4_ group.

To investigate the potential role of PD-1/PD-L1 axis on PE, we used an animal model that mimics some of the clinical characteristics observed in pre-eclamptic patients. Thus, pregnant Sprague Dawley (SD) rats were injected with 125 mg/kg L-NAME on Gestational Day (GD) 13 to establish the pre-eclamptic models. We evaluated systolic blood pressure (SBP), urine protein concentrations, and weight gain of the rats. Table 2 summarizes the differences between treated and untreated rats. L-NAME (−) group had relatively consistent SBP throughout the entire gestation period (SBP of 116.0 ± 1.581 mmHg on GD 16). Rats treated with L-NAME showed increased SBP from 116.6 ± 1.949 mmHg on GD 12 to 138.8 ± 1.643 mmHg on GD16, which was significantly higher than L-NAME (−) group (*P* = 0.000) (Fig. 2A). These findings indicate the administration of L-NAME resulted in hypertension, similar as observed in pre-eclamptic patients.

Our next objective was to determine whether the administration of PD-L1-Fc, an activator of PD-1 pathway, could reverse the PE-like symptoms observed with the administration of L-NAME. Thus, pre-eclamptic rats were treated with PD-L1-Fc (600 μg) on GD16 or saline as negative controls. A third group of the pre-eclamptic rats were treated with 270 mg/kg magnesium sulfate on GD16 as the positive controls. Magnesium sulfate has been considered the first choice in relieving vasospasm and the main choice in the treatment of pre-eclamptic patients. As expected, L-NAME-induced pre-eclamptic rats treated with saline showed continuously increase on SBP from GD14 until delivery. In contrast, the injection of PD-L1-Fc had a major effect on the rats by ameliorating SBP increase. As shown in Fig. 2B, we observed a significant decrease on SBP to normal levels within 6 hours post PD-L1-Fc treatment and remained at normal levels for additional 48 h (Fig. 2A,B). Interestingly, the antihypertensive effect of magnesium sulfate was speedy and transient, while the effect of PD-L1-Fc was steady and sustained (Fig. 2B).

Proteinuria is usually associated with PE and is a marker of renal malfunction. We also evaluated the levels of proteinuria in the rat model and observed that before pregnancy and on GD 10 all groups had the similar levels of urine protein. L-NAME significantly increased the proteinuria levels in pre-eclamptic rats (*P* = 0.000) (Fig. 2C), which indicated the presence of renal dysfunction. Treatment with magnesium failed to reverse this condition; however, after PD-L1-Fc treatment, urinary protein significantly decreased on GD 20 (*P* = 0.030) (Fig. 2C), suggesting that PD-L1-Fc treatment partly restored the renal function.

Apart from the changes of SBP and urinary protein, we also observed less net weight gain in pre-eclamptic rats (*P* = 0.017) (Fig. 2D). Treatment with PD-L1-Fc had a trend on reversing the loss of weight gain although it was not statistically significant (*P* = 0.390) (Fig. 2D).

The effect on renal function observed in pregnant PE rats was confirmed at the tissue level. L-NAME-induced pre-eclamptic rats revealed renal damage, such as increased glomerular abnormalities including cystic-like lesions in the glomeruli and collapse of the glomerular tuft (Fig. 2E). The damage was also observed in liver structure, characterized by widespread necrosis of hepatocytes and fatty accumulation (Fig. 2E). Interestingly, PD-L1-Fc treatment reduced liver and kidney injuries, namely, less necrotic hepatocytes and renal cells, and few diffused fatty changes (Fig. 2E). The analysis of histological scoring revealed that the renal and liver histopathologic injury scores in the L-NAME-administered groups were both significantly elevated (*P* = 0.000, *P* = 0.000, respectively). However, PD-L1-Fc treatment had lower scores compared with other two L-NAME treated groups (Fig. 2F,G). It is worth noting that PD-L1-Fc-treated rats had relatively normal SBP and milder histological injury compared with magnesium sulfate treated rats, albeit similar in body weight gain, suggesting that PD-L1-Fc is more potent than magnesium sulfate in mitigating L-NAME-induced pre-eclamptic symptoms.

### PD-L1-Fc affects the differentiation of Treg and Th17 cell in pre-eclamptic rat models

**Figure 3:**
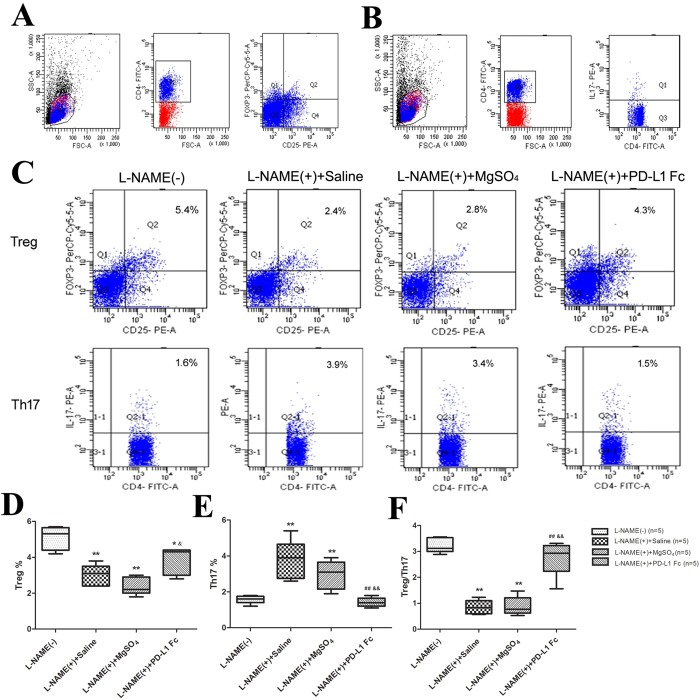
The determinations of rat peripheral Treg and Th17 cells in different groups by flow cytometry. **(A–C**) Gating strategies for Treg cells and Th17 cells used in our study. The percentages of Treg cells (**D**) and Th17 cells (**E**), as well as the Treg/ Th17 cell ratios (**F)** in the four groups were determined. Data are presented as the median (range). *P < 0.05, **P < 0.01 vs. L-NAME (−) group; ^##^*P* < 0.01 vs. L-NAME (+) group; ^&^*P* < 0.05, ^&&^*P* < 0.01 vs. L-NAME (+) +MgSO_4_ group.

As indicated above, both decreased Treg cells and/or increased Th17 cells play a critical role in the pathogenesis of PE^15^,^16^. Thus, we investigated whether the beneficial effect observed with PD-L1-Fc treatment on the PE rat model is associated with changes on Treg/Th17 expression and function. Unlike the definition of Treg cells in humans, in rats we defined Treg cells as CD4^+^ CD25^+^ Foxp3^+^ cells (Fig. 3A). Th17 cells were considered as CD4^+^ IL-17A^+^ cells, similar as in humans (Fig. 3B). Administration of PD-L1-Fc to pre-eclamptic rats on GD16 markedly decreased the percentage of Th17 cells (*P* = 0.008), as well as increased Treg/Th17 cell ratios (*P* = 0.002) (Fig. 3C–F).

### PD-L1-Fc modulates the expression of Foxp3 and RORγt in rat placentas probably via inhibiting PI3K/AKT/mTOR signaling and promoting PTEN expression

**Figure 4:**
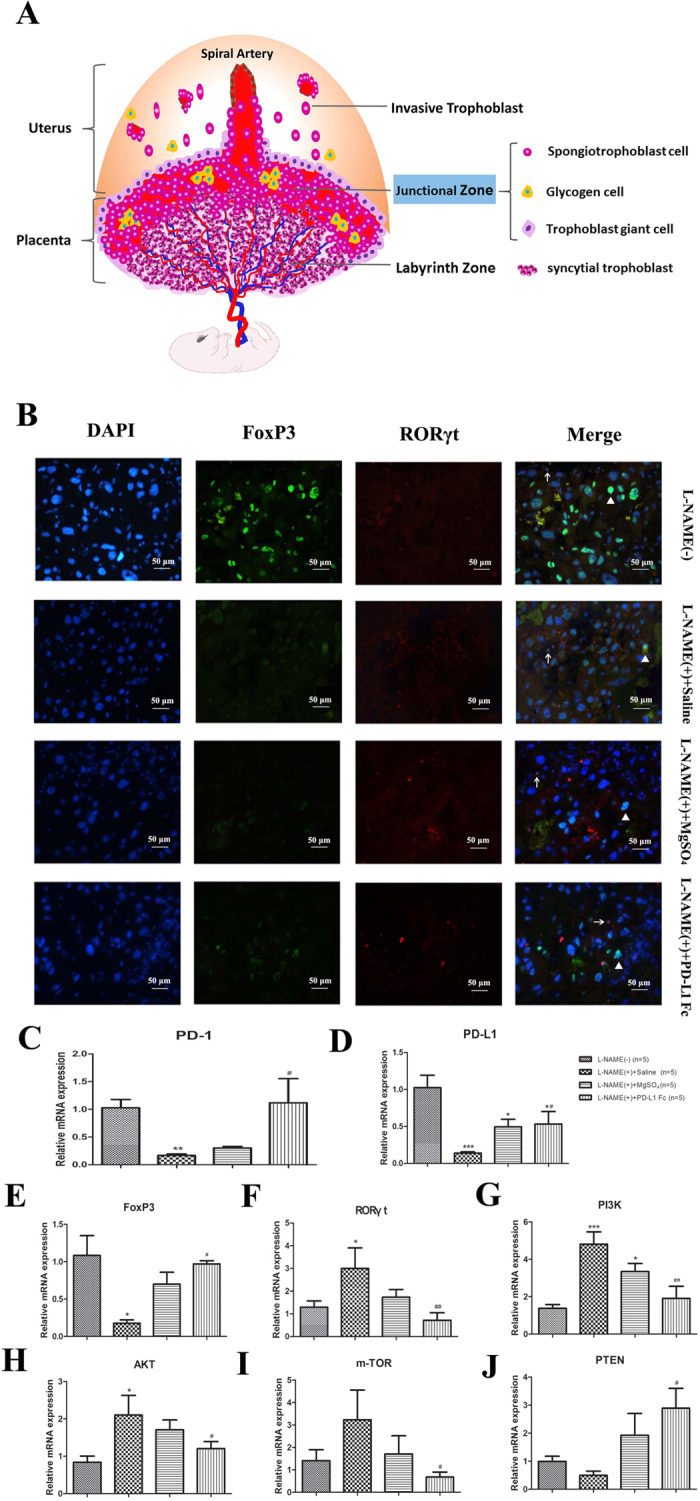
Expression of nuclear transcriptional factors (Foxp3 and RORγt) and the potential signaling molecules in rat placentas. (**A**) The anatomical structure of rat placenta. The rat placenta consists of two regions, the junctional zone (JZ, the interface between maternal and fetal tissues) and the labyrinth zone (LZ, located at the fetal interface). The JZ includes three morphologically distinct compartments: trophoblast giant cells, spongiotrophoblast cells, and glycogen cells. The LZ includes syncytial trophoblast cells. (**B**) The immunofluorescence staining for Foxp3 (green) and RORγt (red) at the JZ in each group. The tissue samples in this part were taken from the maternal side of the JZ in each group and were used for detecting the expression of Foxp3 and RORγt proteins by immunofluorescence staining. After antigen retrieval, the paraffin embedded rat placenta sections were incubated with either rabbit monoclonal antibody to Foxp3 or rabbit monoclonal antibody to RORγt overnight after blocked with BSA. Secondary antibody, goat anti-rabbit IgG (H + L) fluorescein conjugated, was applied and incubated. The nuclei were counterstained by incubating the sections with 4′, 6-diamidino-2-phenylindole (DAPI). The white triangle and the arrow mark the protein expression of Foxp3 and RORγt in the nucleus, respectively. (**C–J**) The mRNA levels of PD-1, PD-L1, Foxp3, RORγt and signaling molecules (PI3K, AKT, m-TOR and PTEN) were determined by RT-PCR in the placentas of the rats in each group. Data are presented as Mean ± SEM. *P < 0.05, **P < 0.01, ***P < 0.001 vs. L-NAME (−) group; ^#^*P* < 0.05, ^##^*P* < 0.01 vs. L-NAME (+) group.

The nuclear transcription factors, Foxp3 and RORγt play crucial roles in the quantity and function of Treg and Th17 cells, respectively, and it has been shown that their expression levels match to the percentages of the corresponding subsets. In order to investigate the potential role of PD-L1-Fc at the maternal-fetal interface, we evaluated the expression of Foxp3 and RORγt in placental samples from the four groups. The rat placenta consists of two regions, the junctional zone (JZ, the interface between maternal and fetal tissues) and the labyrinth zone (LZ, located at the fetal interface) (Fig. 4A). The JZ consists of three morphologically distinct cell types: trophoblast giant cells, spongiotrophoblast cells, and glycogen cells. The LZ includes syncytial trophoblast cells. The protein expression of Foxp3 and RORγt at the maternal-fetal interface in each group were shown in representative placental samples taken from the maternal side of the JZ using immunofluorescence staining (Fig. 4B). Administration of PD-L1-Fc to PE-like rats was associated with the increased expression of Foxp3 and reduced RORγt in the placenta compared to PE-like rats that received vehicle. These findings were confirmed at the mRNA levels as shown by higher expression of Foxp3 (*P* = 0.014) and lower expression of RORγt (*P* = 0.008) in placentas after PD-L1-Fc treatment (Fig. 4E,F). Interestingly, the mRNA levels of PD-1 and PD-L1 increased in the PD-L1-Fc treated groups compared with untreated pre-eclamptic rats (*P* = 0.013, *P* = 0.048, respectively) (Fig. 4C,D).

**Figure 5:**
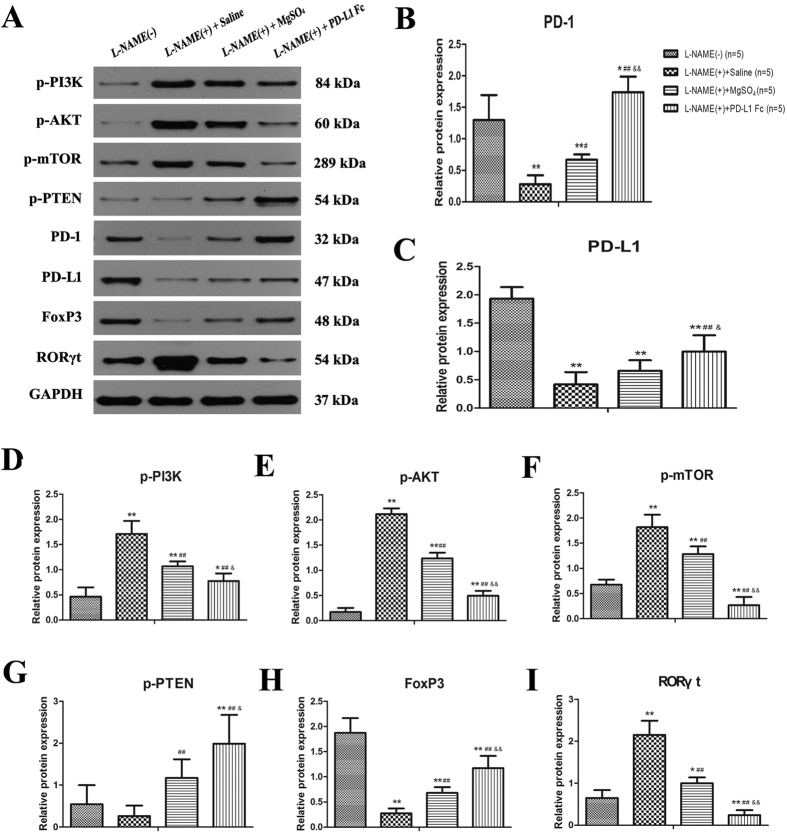
Protein expression of PD-1, PD-L1, Foxp3, RORγt and the potential signaling molecules in rat placentas. The protein levels of PD-1, PD-L1, Foxp3, RORγt and signaling molecules (p-PI3K, p-AKT, p-mTOR and p-PTEN) were determined by western blotting in the placentas of the rats in each group. Data are presented as Mean ± SEM. *P < 0.05, **P < 0.01 vs. L-NAME (−) group; ^#^*P* < 0.05, ^##^*P* < 0.01 vs. L-NAME (+) group; ^&^*P* < 0.05, ^&&^*P* < 0.01 vs. L-NAME (+) + MgSO_4_ group.

In order to elucidate the protective effect of PD-L1-Fc more clearly, its potential mechanism was further investigated. It has been reported that the PD-1/PD-L1 pathway limits T-cell stimulation and promotes the differentiation and maintenance of Foxp3^+^ Treg cells by blocking the AKT/mTOR pathway and augmenting PTEN expression. In line with former reports, we proved that the mRNA expression of PI3K/AKT/mTOR declined and that of PTEN increased in the placenta after PD-L1-Fc administration (Fig. 4G–J). Furthermore, the phosphorylation levels of P13K, Akt, mTOR and PTEN, and the protein expression levels of PD-1, PD-L1, Foxp3 and RORγt were detected. And we got the consistent results as those of the corresponding mRNA expression (Fig. 5). These findings suggested that PD-1/ PD-L1 pathway was abnormal in pre-eclamptic rat placentas, and PD-L1-Fc modulated the expression of Foxp3 and RORγt probably via inhibiting PI3K/AKT/mTOR signaling and promoting PTEN expression.

### PD-L1-Fc protects fetuses from abnormal development in pre-eclamptic rats

PE is among the leading causes of fetal growth restriction (FGR) and results from the impairment of maternal-fetal exchanges^33^. To evaluate whether PD-L1-Fc plays a protective role on the fetuses, we evaluated the development of fetus and placenta, as well as the percentage of fetal resorption in PD-L1-Fc-treated pre-eclamptic rats on GD21. The data of the fetal size and placental size in L-NAME treated groups were all from un-resorpted fetuses.

**Figure 6:**
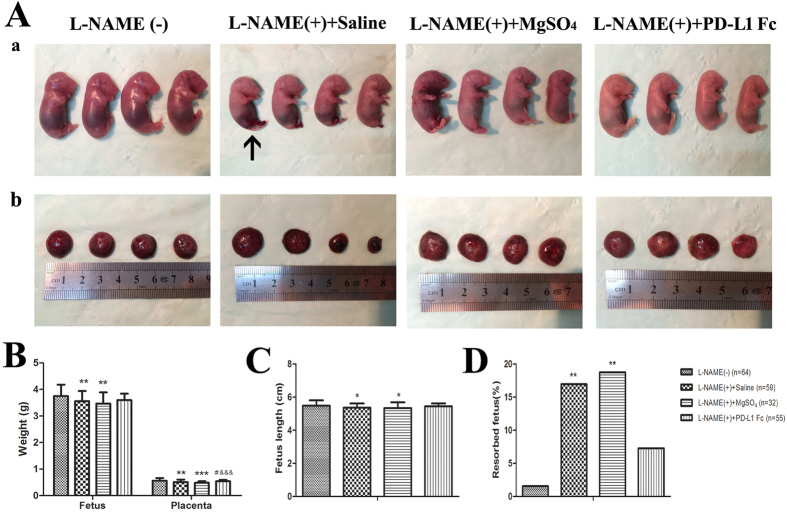
Effects of PD-L1-Fc on rat fetal outcomes. (**A**) Representative pictures of fetal congenital malformation and small placentas were observed in pre-eclamptic rats. **(B–D**) Fetus weight, placenta weight, fetus length and the rates of resorbed fetus in each group were measured. Data are presented as Mean ± SEM. Data in **(D**) are analyzed by Chi-square test. *P < 0.05, **P < 0.01, ***P < 0.001 vs. L-NAME (−) group; ^#^*P* < 0.05 vs. L-NAME (+) group; ^&&&^*P* < 0.001 vs. L-NAME (+) +MgSO_4_ group.

Pups delivered from PE-like rats are characterized by FGR, malformation, lower fetus weight and shorter fetus length (Fig. 6A–C). In addition, PE-like rats have a higher percentage of fetal resorption than in healthy pregnant rats (*P* = 0.003) (Fig. 6D). Treatment with PD-L1-Fc had major effects on fetus outcomes as shown by increased trend of fetal weight and length, and decrease in the percentage of fetal resorption (Fig. 6B–D). Apart from the effect on fetal well-being, we also observed a protective effect on the placenta where treatment with PD-L1-Fc reversed the damage observed in the PE-like rats, mainly associated with placental weight (Fig. 6B).

## Discussion

In the present study, we demonstrated for the first time that altered PD-1/PD-L1 axis may be associated with Treg/Th17 imbalance in women with PE and the use of PD-L1-Fc in an L-NAME-induced PE-like rat models proved to be more effective than magnesium sulfate in protecting the pregnancy and reversing the Treg/Th17 imbalance. Furthermore, we demonstrate that this modulatory effect is mediated via inhibiting PI3K/AKT/mTOR signaling and promoting PTEN expression. The PD-1/PD-L1 pathway has been reported to be crucial for the induction of peripheral immune tolerance and the establishment of therapeutic strategy for autoimmune diseases. Engagement of PD-L1 with its receptor, PD-1 on T cells leads to the promotion of Treg development and function and down-regulation of T-cell responses. The interactions between PD-1 and PD-L1 participate in the maintenance of pregnancy partly by regulating Treg- and Th17-immunity^29^,^30^.

In line with earlier reports^15^,^34^,^35^,^36^,^37^,^38^, we confirmed that both the deficiency of Treg quantity and excess activation of Th17 cells were associated with PE in humans. However, the leading factors to the Treg/Th17 imbalance have not been fully elucidated in PE.

Several other factors might account for this, while the roles of certain co-inhibitor receptors (such as PD-1, CTLA-4, Tim-3 and CD244) in the development of the maternal systemic inflammatory response have rarely been studied in PE. Among all of the inhibitory receptors expressed in T cells, PD-1 is considered to be the dominant responsive inhibitory receptor^39^. PD-1 cross-linking with PD-L1 was crucial for the prevention of chronic rejection in a heart transplant model^40^ as well as for the maintenance of tolerance at the utero-placental interface^41^. PD-1 deficient mice are more likely to suffer from autoimmune diseases than the wild type. Therefore, it’s rational to assume that abnormal PD-1/PD-L1 axis might contribute to immune disorder. Interestingly, we observed significant changes on the expression levels of PD-1 and PD-L1 on Treg and Th17 cells in pre-eclamptic patients compared to normal controls. These differences were cell specific; thus, we found increased PD-1 expression on Treg cells, while Th17 cells had decreased PD-1 and increased PD-L1. These findings are in agreement with the results reported by Toldi *et al.*^42^, who suggested that higher PD-1^+^ Treg percentages might account for the reduction of Treg cells in PE. Therefore, dysfunctional PD-1/PD-L1 pathway may account for the Treg/Th17 imbalance observed in PE patients.

It has been shown that the PD-L1-Fc significantly alleviates symptoms and suppresses disease progress in murine models suffering from auto-immune disorders or GVHD by promoting the development of Treg cells and inhibiting the differentiation of Th17 cells^24^,^25^,^31^. Thus, PD-L1-Fc has been considered a rational target for autoimmune disease therapy. Furthermore, it has been shown that PD-L1 blockade with anti-PD-L1 neutralizing antibody increased embryo resorption rate and reduced litter sizes in early pregnancy^28^. In order to study the potential effect of PD-L1-Fc in PE *in vivo*, we successfully set up a pre-eclamptic rat model by subcutaneously injection of L-NAME^33^,^43^. Pregnant rats develop higher SBP and proteinuria, the major clinical characteristics observed in PE patients^33^,^43^. As expected, we found that PD-L1-Fc treatment ameliorated PE-like symptoms by decreasing SBP and alleviating renal and hepatic lesions, indicating the potential protective role of PD-L1-Fc in PE. The high SBP of the PE-like rats could decrease to normal levels and maintain at low levels with the administration of PD-L1-Fc. As the effect of the therapy diminished (elimination of the protein from the system), we observed a steady increase on SBP, suggesting the need to maintain the constant levels of PD-L1-Fc in order to prevent PE-like symptoms. Additional studies are needed to evaluate the optimal doses, frequency and potential toxicity of PD-L1-Fc therapy.

This effect on the PE-rat model was confirmed to be mediated by PD-1/Treg/Th17 axis since. PD-L1-Fc treatment suppress pathogenic Th17 cells and had a trend on increasing the percentage of protective Treg cells. In addition we found that this effect is not limited to circulating Treg/Th17 cells, but also at the local placenta. PD-L1-Fc administration to PE-rats was associated with increased expression of Foxp3 and reduced expression of RORγt in the placenta compared with control untreated pre-eclamptic rats, showing a suppressive effect on both systemic and local inflammation.

PD-L1-Fc administration has been shown to have a preferential suppression of pathogenic Th17 cells in animal models suffering from colitis or arthritis^24^,^31^. In addition, engagement of PD-L1-Fc promoted Treg development in the presence of TGF-β^44^. PD-L1 blockade reversed the inhibition of mature Th17 cells and the promotion of Treg cells at the maternal-fetal interface^28^. It is rational to assume that Treg/Th17 imbalance in PE might be reversed by PD-L1-Fc treatment.

PD-1 is considered to be a marker for exhausted T cells^45^,^46^. Engagement with PD-L1, PD-1 suppresses nuclear factor kappa B (NF-κB)-mediated production of cytokines by inhibiting PI3K activity and downstream activation of AKT/m-TOR^47^. While in the presence of anti-CD3 and TGF-β, PD-L1-Fc induces a profound increase in the *de novo* generation of CD4^+^ Foxp3^+^ Treg cells from naïve CD4^+^ T cells^44^. Moreover, PD-L1-Fc also enhances Foxp3 expression and suppresses function of established Treg cells^44^. In mechanistic studies, it has been proven that PD-L1-Fc induces Treg cells from naïve T cells by attenuation of AKT-mTOR signaling and concomitant up-regulation of PTEN^31^,^44^. These observations are consistent with our data that PD-L1-Fc treatment promotes Foxp3 expression and suppresses RORγt expression, accompanied by reduced level of AKT/mTOR and elevated PTEN expression in the placenta. Namely, PD-L1-Fc reversed Treg/Th17 imbalance by inhibiting PI3K/AKT/m-TOR signaling and enhancing PTEN expression in pre-eclamptic rats. In addition, PD-1 and PD-L1 were expressed in rat placentas. It has been demonstrated that PD-L1 was consistently expressed by the placenta throughout pregnancy in humans, and remained higher in the second and third trimester of pregnancy in order to facilitate protection of fetal cells against activated maternal leukocytes^26^. Therefore, lower PD-L1 expression might affect this delicate balance altering the maternal responses against fetal cells as observed in the pre-eclamptic rats.

In our study, PD-L1-Fc administration increased PD-L1 expression in PE-like rats, which might also participate in the induction of maternal immune homeostasis. It’s well known that PD-1 is expressed in immune cells, especially the lymphocytes. Apart from immune cells, PD-1 is reported expressed in tumor cells, such as human melanomas^48^. Furthermore, PD-1 overexpression promotes tumor growth, while inhibition of melanoma-PD-1 reduced tumor growth, independently of adaptive immunity *in vivo*^48^. It is well known that the placenta functions as a tumor in many ways, such as proliferation invasion and immune regulation. In our study, PD-1 was observed in rat placenta and decreased in PE-like rats while PD-1 expression increased in PD-L1-Fc treated rats. It is rational to assume that PD-1 might promote placenta growth and maturation by engagement with PD-L1 in a similar way as tumor cells. Actually, this hypothesis is supported by our data showing that, the weight of placentas in the PD-L1-Fc treated group was similar to that in healthy pregnant rats. This suggests that immunotherapy with PD-L1-Fc may produce an effect on placenta growth that is separate from their effect on the immune response.

Apart from the effect on the mother, PE poses a great threat to the well-being of fetuses, such as FGR^49^. FGR occurs when the fetus fails to achieve its genetically predetermined growth potential^50^. Although the precise mechanism leading to the development of FGR remains unknown, there is evidence that these complications are associated with an aberrant maternal inflammatory response^51^. Women afflicted by FGR exhibit a heightened inflammatory state: proinflammatory cytokines and chemokines are elevated systemically and locally in the placenta^51^,^52^. It is well established that FGR most commonly results from utero-placental insufficiency, such as PE, which is a later clinical manifestation of poor placentation and placental ischemia^53^. In the current study, we observed fetal malformation in PE-like rats, such as hind limb deformation. This is consistent with previously published reports^33^. However, no fetal malformations were observed in the PD-L1-Fc-treated rats. Likewise, we observed lower fetus weight and fetus length in PE-like rats; which concur with published reports^54^. Partial reversal of growth retardation was observed in the PD-L1-Fc treated group where the weight and length of the fetuses were similar with that of fetuses from the healthy pregnant rats. We also demonstrated that placentas from PE-like rats differed from placentas from healthy pregnancies. In the PE-like models, placental weight was lower. This was in line with the studies conducted by Ramesar and Ma^55^,^56^, who also found a decrease in the placental weight in SD rat treatment with L-NAME. In addition, a higher percentage of fetal resorption in PE-like models was observed in our and others’ studies^57^. PD-L1-Fc decreased fetal resorption in PE-like rat model. The decrease in the placental weight suggested that insufficient placentation decreased placental blood flow and fetal oxygen supply led to lower fetal weight and lower fetal length in PE. All this abnormal developmental effects can be reversed with PD-L1-Fc further supporting the critical role of PD-1/PD-L1 in pregnancy.

Taken together, our findings provide new insight into the function of PD-1/PD-L1 pathway during pregnancy by maintain a functional balance between Treg/Th17 cells. Changes in the expression and function of PD-1/PD-L1 axis might be responsible for the imbalance of Treg/Th17 cells and the malignant phenotype observed in PE. PD-L1-Fc administration proved to have a protective effect, both on the mother and fetus, *in vivo* by stimulating the PD-1/PD-L1 T-cell co-inhibitory pathway and eventually reversing Treg/Th17 imbalance. This effect is mediated by inhibition of PI3K/AKT/m-TOR signaling and enhancement of PTEN expression. In addition, our results suggested the PD-L1-Fc might be beneficial for pregnancy by promoting placenta growth independently of adaptive immunity. As such, our study shed new light on the mechanisms underlying PE, providing new pathways that could be targeted for treatment of this disorder.

## Methods

### Human experiment

#### Subjects

This study was reviewed and approved by Huazhong University of Science and Technology Clinical Trial Ethics Committee. All methods were carried out in accordance with the approved guidelines and regulations. A written informed consent was obtained from each participant prior to entering the study. All the study subjects were recruited from the Department of Obstetrics and Gynecology at Tongji Hospital, Wuhan, China. A total of 40 women were recruited including 22 women with NP and 18 with PE. The clinical characteristics of the two groups are presented in Table 1. The definition of PE has been described elsewhere^58^. All pregnancies in this study were singleton gestations. None of the participants was affected by pre-existing clinical disorders or had active labor at the time of enrollment and blood sampling.

#### Blood sampling

Peripheral blood samples were collected into heparinized tubes and peripheral blood mononuclear cells (PBMC) were isolated by the density centrifugation technique and were collected, washed, and kept in a liquid-nitrogen filled container for cryopreservation until use.

#### Flow cytometric analysis

Thawed PBMC were washed and re-suspended, and the cell viability was measured by Trypan blue staining. A total of 2 × 10^6^ cells were stained with anti-human CD4-FITC, anti-human CD25-PE, anti-human CD127-PE-Cy5, anti-human PD-1-APC and anti-human PD-L1-PE-Cy7 for surface antigens (eBioscience, San Diego, CA, USA), in accordance with the manufacturer’s instructions. For intracellular cytokine detection, 2 × 10^6^ cells were stimulated with 2 μL of Cell Stimulation Cocktail and 2 μL of Protein Transport Inhibitor Cocktail (eBioscience, San Diego, CA, USA) for 5 h. Collected cells were first stained with fluorescein-labelled mAbs for surface antigens, followed by fixation using IC Fixation buffer (eBioscience, San Diego, CA, USA). Then, the cells were stained with anti-human IL-17A-PE (eBioscience, San Diego, CA, USA) in an appropriate volume of 1 × Permeabilization Buffer (eBioscience, San Diego, CA, USA) for 25 min. Finally, the cells were re-suspended in 300 μL of PBS for subsequent flow cytometric analysis. All data was acquired on FACScalibur (BD Biosciences, San Jose, CA), and processed using the CellQuest program (Becton Dickinson, Franklin Lakes, NJ).

### Animal experiment

#### Experimental animals

Animal care and use were reviewed and approved by the Institutional Animal Care and Use Committee of Tongji Medical College, Huazhong University of Science and Technology, Wuhan, China. All procedures were carried out in accordance with the approved guidelines of the Institutional Animal Care and Use Committee of Tongji Medical College. Sprague Dawley (SD) rats were purchased from the Animal Centre of Tongji Medical College. Female rats weighed 200–250 g were raised in a light- and humidity-controlled room with free access to food and water and allowed to acclimatize for one week and then they were placed in mating cages with male SD rats. The day on which pregnancy was confirmed by the presence of vaginal spermatozoa was designated as Gestational Day (GD) 1.

#### Groups and treatments

From GD13 to GD19, 125 mg/kg body weight (BW) L-NAME (Sigma, St.Louis, MO) was subcutaneously injected every day to induce PE-like models. The pregnant rats were separated into four groups and treated as follows: (I) control group, healthy pregnant rats without any treatment, namely, L-NAME (−) group (n = 5); (II) L-NAME(+) + saline group, treated with 270 mg/kg BW saline on GD 16 by subcutaneous injection (n = 5); (III) L-NAME(+) +MgSO_4_ group, treated with 270 mg/kg BW magnesium sulfate on GD 16 by subcutaneous injection (n = 5); (IV) L-NAME(+) +PD-L1-Fc (Chimerigen, San Diego, CA, USA) group, treated with 600 μg PD-L1-Fc on GD 16 by subcutaneously injection (n = 5).

#### Measurement of blood pressure, 24-h urinary protein and body weight gain

The systolic blood pressure (SBP) of each rat was measured prior to pregnancy and every other day (08:00 am–10:00 am) from GD 2 to GD 20 using the ZH-HX-Z animal non-invasive blood pressure measuring system (Zhenghua Biological Instrument and Equipment Limited Corporation, Anhui, China). The SBP was assessed continuously 5 times, in which 3 continuous values of variation of less than 6 mmHg were averaged to define maternal SBP. Moreover, dynamic blood pressure was monitored in the last two groups on GD16 until sacrificed. Blood pressure, especially the SBP, increased by no less than 20 mmHg after L-NAME treatment was the key criteria in evaluating whether it’s truly a pre-eclamptic model. The urine of each rat was collected on three certain days (10:00 am–10:00 am on next day), namely, GD10 (three days before L-NAME treatment), GD17 (next day after treatment) and GD20 (the day before sacrifice) individually in metabolic cages. Urine protein level was measured using a BCA protein assay kit (Pierce Biotechnology, Rockford, IL). In addition, we measured the BW of each rat every other day from GD 2 to GD 21. Then the net BW gain throughout pregnancy (the difference between GD21 and GD2 BW), after GD12 (the difference between GD21 and GD12 BW) and after GD16 (the difference between GD21 and GD16 BW) were calculated.

#### Sample collection

At GD21, the animals were sacrificed and spleens were resected for flow cytometry. The kidneys and livers were harvested for histopathologic analysis. Some placentas were fixed in 4% paraformaldehyde and embedded in paraffin for immunofluorescence staining. The remaining placentas were kept in −80 °C refrigerator after being sub-packaged in the EP tubes prepared for real time PCR. The following parameters of pregnancy outcome were evaluated: placenta weight, embryo resorption rate, fetus weight and length.

#### Histological analysis

The 4-μm-thick paraffin sections of rat kidney and liver tissues were stained with haematoxylin and eosin (H&E) according to the standard H&E protocol. Sections were examined by a qualified and blinded pathologist to evaluate the degree of pathological changes. A semi-quantitative scale was used in evaluating the morphological characteristics of kidney as suggested by Hamer’s standard *et al.*^59^, and the samples were scored for liver histological damages in 20 random visual fields in accordance with Suzuki’s criteria^60^.

#### Flow cytometry for Treg and Th17 cells in rat spleen

The rat spleen tissues were gently teased to release cells in PBS. The splenic mononuclear cells were isolated by density centrifugation technique. Isolated splenic mononuclear cells were adjusted to 1 × 10^7^ cells/mL and stained with anti-rat CD4-FITC, and/or anti-rat CD25-PE (eBioscience, San Diego, CA, USA) per 100 μL. For Th17 analysis, cells were stimulated and incubated with Cell Stimulation Cocktail (plus Protein Transport Inhibitor) (500 x) (eBioscience, San Diego, CA, USA) for 5 h. Then the cells were fixed and premeabilised for 30 min at room temperature at dark with the Fixation/Permeabilization Diluent (eBioscience, San Diego, CA, USA). Finally, the cells were stained with anti-mouse/rat Foxp3 PE-Cy5.5 (eBioscience, San Diego, CA, USA) or anti-Mouse/Rat IL-17A PE (eBioscience, San Diego, CA, USA). The data were performed and processed in the same way as mentioned before.

#### Quantitative real-time PCR (qRT-PCR)

Total RNA was extracted and purified using TRIzol reagent (Invitrogen, Carlsbad, CA, USA) and an equal amount of total RNA (1 μg) was used for cDNA synthesis (Takara Bio, Shiga, Japan). Primer sets used in this study (shown in Supplementary Table S1) were designed using Primer-BLAST software. 2 μl cDNA was subjected to qRT-PCR amplification analysis using SYBR Green PCR mix (Applied Biosystems). The qRT-PCR parameters were: step one, one cycle at 95 °C for 30 sec; step two, 40 cycles at 95 °C for 5 sec, 60 °C for 30 sec, and 72 °C for 30 sec; step three, 95 °C for 15 sec, 60 °C for 60 sec, and 95 °C for 15 sec (LightCycler^®^ 96 System, Roche, Germany). The amount of target relative to a calibrator was computed by 2^−ΔΔCT^, and β- action was used for normalization.

#### Immunofluorescence staining

After antigen retrieval, the paraffin embedded rat placental tissue sections were blocked with 5% BSA for 20 min, and incubated with either rabbit monoclonal antibody to RORγt (Abcam, Cambridge, MA; dilution 1:100) or rabbit monoclonal antibody to Foxp3 (Abcam, Cambridge, MA; dilution 1:300) overnight at 4 °C after rejection to the BSA solution. Secondary antibody, goat anti-rabbit IgG (H + L) fluorescein conjugated (CHEMICON, Temecula, CA, 1:50), was applied and incubated 30 minutes at 37 °C in the dark. The nuclei were counterstained by incubating the sections for 5 min with 4′, 6-diamidino-2-phenylindole (DAPI). Slides were washed with PBS, mounted with Antifade. Images were acquired and analyzed with a microscope (Leica AF CTR6500HS).

#### Western blotting analysis

Following SDS/PAGE, transfer and blocking, the PVDF blots were incubated with primary antibodies (shown in Supplementary Table S2). The membranes were washed and incubated with a secondary antibody (shown in Supplementary Table S3), followed by ECL-detection. Relative protein levels were quantified by scanning densitometry and analyzed by the Image J software (National Institutes of Health, Bethesda, MD).

### Statistical analysis

Statistical analysis was performed using Statistical Package for Social Science (SPSS) for Windows (Version 18.0 software, SPSS Inc., Chicago, IL, USA) and the GraphPad Prism software, version 5 (GraphPad). Differences between Treg and Th17 cells were analyzed using Student’s *t*-test or the Mann-Whitney *U*-test as applicable. The differences between multiple groups were analyzed by one-way ANOVA and Chi-square test. The P-value less than 0.05 was considered significant.

## Additional Information

**How to cite this article**: Tian, M. *et al.* The PD-1/PD-L1 inhibitory pathway is altered in pre-eclampsia and regulates T cell responses in pre-eclamptic rats. *Sci. Rep.* **6**, 27683; doi: 10.1038/srep27683 (2016).

## Supplementary Material

Supplementary Information

## Figures and Tables

**Table 1:** Clinical characteristics of all participants (n = 40).

Characteristics	Normal pregnancy (n = 22)	Pre-eclampsia (n = 18)	*P* value
Age (years)	29.6 ± 0.8	31.5 ± 1.3	0.214
Gestational age (weeks)	37.6 ± 0.3	36.5 ± 0.5	0.089
Gravidity	2.0 ± 0.2	1.9 ± 0.3	0.852
Parity	0.3 ± 0.1	0.3 ± 0.1	0.977
Systolic blood pressure (mmHg)	122.5 ± 1.2	153.6 ± 3.4	0.000
Diastolic blood pressure (mmHg)	78.0 ± 1.3	103.1 ± 3.0	0.000
Proteinuria	–	++	0.000
Creatinine (*μ*mol/L)	49.7 ± 2.0	68.2 ± 5.8	0.000
Uric acid (*μ*mol/L)	284.5 ± 15.6	406.9 ± 21.1	0.000

Data are presented as Mean + SEM.

**Table 2:** Effect of PD-L1-Fc on L-NAME-induced PE-like symptoms in rat models.

	L-NAME(−) (n = 5)	L-NAME(+)+Saline (n = 5)	L-NAME(+)+MgSO_4_ (n = 5)	L-NAME(+)+PD-L1-Fc (n = 5)
SBP on D12 (mmHg)	113.8 ± 5.586	116.6 ± 1.949	119.0 ± 3.082	115.8 ± 3.564
SBP on D14 (mmHg)	116.2 ± 3.271	133.0 ± 2.449	133.2 ± 2.950	134.6 ± 3.130
SBP on D16 pre-treatment (mmHg)	116.0 ± 1.581	138.8 ± 1.643	136.6 ± 2.302	139.8 ± 2.775
SBP on D16–1 h post-treatment (mmHg)			138.4 ± 1.517	138.2 ± 1.924
SBP on D16– 2 h post-treatment (mmHg)			120.6 ± 2.881**	138.2 ± 2.168
SBP on D16– 4 h post-treatment (mmHg)				134.8 ± 1.304
SBP on D16– 6 h post-treatment (mmHg)				124.6 ± 1.949**
SBP on D17 (mmHg)			139.2 ± 1.643	121.8 ± 2.775**
SBP on D18 (mmHg)	116.6 ± 3.209	140.6 ± 2.881	139.0 ± 1.871	121.0 ± 2.550**
SBP on D19 (mmHg)				131.4 ± 1.517
SBP on D20 (mmHg)	115.2 ± 5.848	142.0 ± 2.121	140.0 ± 2.828	133.8 ± 1.643
Urine protein on D10 (mg/24 h)	3.144 ± 0.686	2.962 ± 0.248	2.414 ± 0.238	2.688 ± 0.277
Urine protein on D17 (mg/24 h)	3.206 ± 0.617	5.168 ± 0.624	4.662 ± 0.396	3.940 ± 0.260
Urine protein on D20 (mg/24 h)	3.234 ± 0.733	6.242 ± 0.663	5.874 ± 0.404	5.182 ± 0.097
Weight gain throughout pregnancy (g)	109.960 ± 19.468	77.340 ± 22.167	63.800 ± 23.527	80.600 ± 9.318
Weight gain after D12 (g)	84.100 ± 17.590	58.980 ± 23.219	31.920 ± 16.479	61.500 ± 9.368
Weight gain after D16 (g)	54.420 ± 12.677	36.100 ± 16.393	31.500 ± 6.691	47.320 ± 13.074

Data are analyzed by One-way ANOVA, and presented as Mean+ SEM. ^**^P < 0.01 vs. SBP on D16 pre-treatment.
