# Supplementary material for: The PD-1/PD-L1 inhibitory pathway is altered in pre-eclampsia and regulates T cell responses in pre-eclamptic rats
Source: Sci Rep. 2016 Jun 9;6:27683. doi: 10.1038/srep27683 (PMC4899740; doi:10.1038/srep27683)
Supplement: Supplementary Information [file srep27683-s1.pdf]

Supplementary Information

Title: The PD-1/PD-L1 inhibitory pathway is altered in pre-eclampsia and regulates T cell responses in pre-eclamptic rats

Author list: Mei Tian[1*], Yonghong Zhang[1*], Zhaozhao Liu[1*], Guoqiang Sun[2], Gil Mor[3] & Aihua Liao[1]

Affiliations: [1]Family Planning Research Institute, Center for Reproductive Medicine, Tongji Medical College, Huazhong University of Science and Technology, Wuhan 430030, P.R. China, [2]Department of Obstetrics and Gynecology, Maternal and Child Health Hospital of Hubei province, Wuhan, P.R. China, [3]Department of Obstetrics, Gynecology & Reproductive Sciences, Division of Reproductive Sciences, Yale University School of Medicine, New Haven, CT 06520, USA.

* These authors contributed equally to this work.

Correspondence and requests for materials should be addressed to A.L. (aihua_liao@sina.com)

Supplementary Table S1

Primer sequences for quantitative real-time RT-PCR

Target	Forward primer (5' to 3')	Reverse primer (3' to 5')
β-actin	CTGAACCCTAAGGCCAACCG	GACCAGAGGCATACAGGGACAA
PD-1	TCTCGCTGCCTTCTGCTCAACA	GTCTTCTCTCGTCCCTGAAAGTCCA
PD-L1	CAACGGGAAACCCATCACCA	ACGCCAGTAGACTCCACGACAT
PI3K	CTTGCCTCCATTCACCACCTCT	GCCTCTAATCTTCTCCCTCTCCTTC
AKT	TGTCTCGTGAGCGCGTGTTTT	CCGTTATCTTGATGTGCCCGTC
m-TOR	GGCTTCTGAAGATGCTGTCC	GAGTTCGAAGGGCAAGAGTG
PTEN	AAACAGTAGAGGAGCCATCAAAT	TCAGAGTCAGTGGTGTCAGAATA
Foxp3	GGTTCACACGCATGTTCGCCTA	CACACTGCTCCCTTCTCACTCTCC
RORγt	CGGGCCTACAATGCTGACA	GCCACCGTATTTGCCTTCAA

Supplementary Table S2

Primary antibodies used in western blotting

Antibody	Company	Origin	Dilution
GAPDH	Abcam	Rabbit	1:10000
p-PI3K	Abcam	Rabbit	1:1000
p-AKT	CST	Rabbit	1:1000
p-mTOR	Abcam	Rabbit	1:1000
p-PTEN	CST	Rabbit	1:1000
PD-1	Bioss	Rabbit	1:500
PD-L1	Santa Cruz	Rabbit	1:500
Foxp3	Abcam	Mouse	1:1000
RORγt	Bioss	Rabbit	1:500

Supplementary Table S3

Secondary antibodies used in western blotting

Antibody	Company	Origin	Dilution
HRP-Goat anti Rabbit	KPL	Rabbit	1:10000
HRP-Goat anti Mouse	KPL	Rabbit	1:10000

Supplementary Figure S1

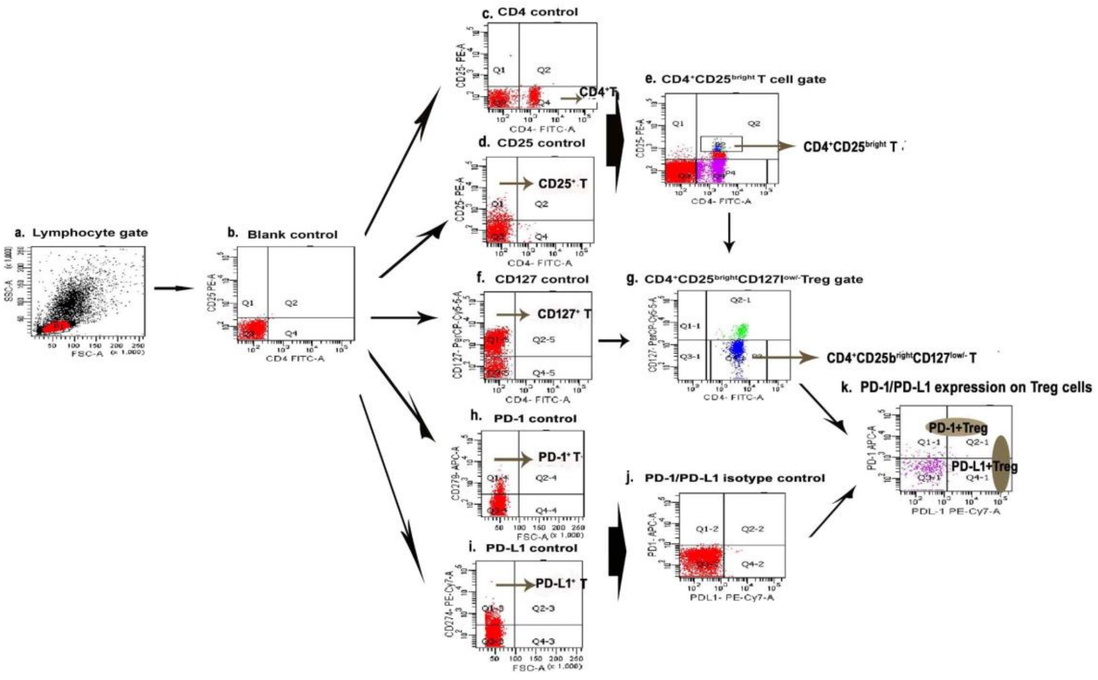

Gating strategy for PD-1 and PD-L1 expression on Treg cells For analysis, the PBMCs were gated on lymphocytes (based on forward and side light scatter) to identify the lymphocyte gate (a). According to the blank control (b) and single standard controls for FITC-CD4 (c) and PE-CD25 (d) mAbs, the $CD4^{+}CD25^{bright}$ T cell gate (e) was determined. Then the gate of $CD4^{+}CD25^{bright}CD127^{low/-}$ Treg cells (g) was defined in terms of the single standard control for Percy-Cy5.5-CD127 (f) mAbs. On the other hand, the separation line about positive or negative of PD-1 and PD-L1 expression were determined according to the blank control (b), single standard controls for APC-PD-1 mAbs （IgG_1 κ） (h) and PE-Cy7-PD-L1 mAbs （IgG_1 κ） (i) and isotype controls for PD-1 （mouse IgG_1κ isotype control APC） and PD-L1 （mouse IgG_1κ isotype control PE-Cy7） (j). Finally, the levels of PD-1 and PD-L1 expression on Treg cells were determined: PD-1^{+}Treg (Q1-1+Q2-1) and PD-$L1^{+}$Treg (Q2-1+Q4-1) (k).

Supplementary Figure S2

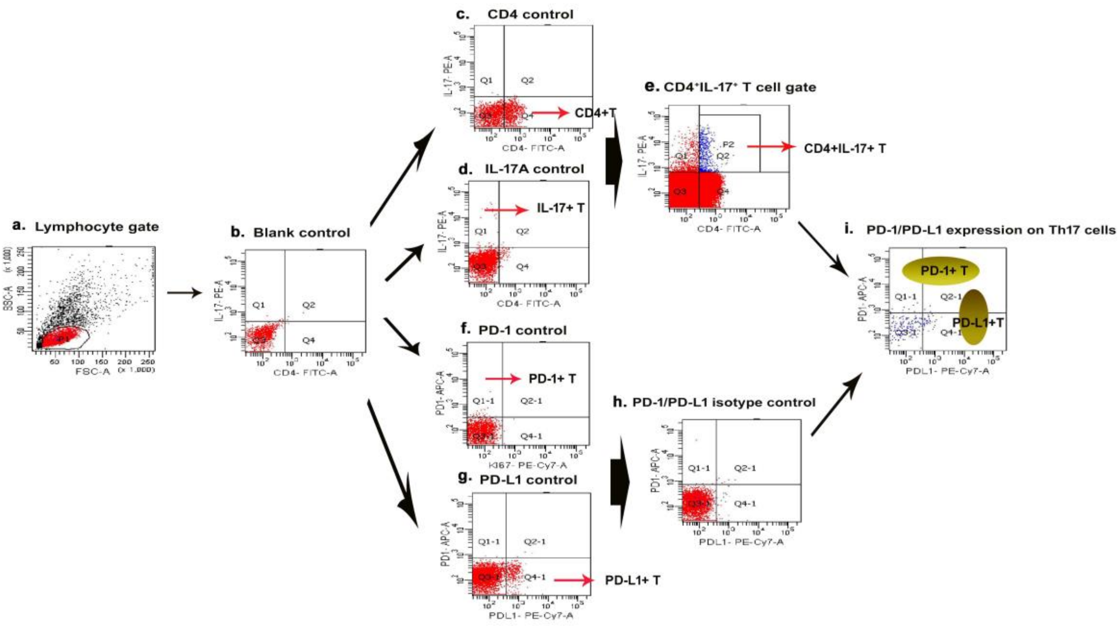

Gating strategy for PD-1 and PD-L1 expression on Th17 cells. For analysis, the PBMCs were gated on lymphocytes (based on forward and side light scatter) to identify the lymphocyte gate (a). According to the blank control (b) and single standard controls for FITC-CD4 (c) and PE-IL-17A (d) mAbs, the $CD4^{+}IL\text{-}17^{+}$ T cell gate (e) was determined. On the other hand, the separation line about positive or negative of PD-1 and PD-L1 expression were determined according to the blank control (b), single standard controls for APC-PD-1 mAbs（IgG1 κ）(f) and PE-Cy7-PD-L1 mAbs （IgG_1 κ）(g) and isotype controls for PD-1 （mouse IgG_1κ isotype control APC）and PD-L1 （mouse IgG_1κ isotype control PE-Cy7）(h). Finally, the levels of PD-1 and PD-L1 expression on Treg cells were determined: $PD\text{-}1^{+}$Th17 (Q1-1+Q2-1) and $PD\text{-}L1^{+}$Th17 (Q2-1+Q4-1) (i).
